# Differential effects of sex on tuberculosis location and severity across the lifespan

**DOI:** 10.1038/s41598-023-33245-5

**Published:** 2023-04-13

**Authors:** Jinsoo Min, Jae Seuk Park, Hyung Woo Kim, Yousang Ko, Jee Youn Oh, Yun-Jeong Jeong, Ju Ock Na, Sun-Jung Kwon, Kang Hyeon Choe, Won-Yeon Lee, Sung-Soon Lee, Ju Sang Kim, Hyeon-Kyoung Koo

**Affiliations:** 1 Division of Pulmonary and Critical Care Medicine, Department of Internal Medicine, Seoul St. Mary’s Hospital, College of Medicine, The Catholic University of Korea, Seoul, Republic of Korea; 2 Division of Pulmonary Medicine, Department of Internal Medicine, Dankook University College of Medicine, Cheonan, Republic of Korea; 3 Division of Pulmonary and Critical Care Medicine, Department of Internal Medicine, Incheon St. Mary’s Hospital, College of Medicine, The Catholic University of Korea, Seoul, Republic of Korea; 4 Division of Pulmonary, Allergy and Critical Care Medicine, Department of Internal Medicine, Kangdong Sacred Heart Hospital, Hallym University College of Medicine, Seoul, Republic of Korea; 5 Division of Pulmonary, Allergy, and Critical Care Medicine, Department of Internal Medicine, Korea University Guro Hospital, Korea University College of Medicine, Seoul, Republic of Korea; 6 Division of Pulmonary and Critical Care Medicine, Department of Internal Medicine, Dongguk University Ilsan Hospital, Dongguk University College of Medicine, Goyang, Republic of Korea; 7 Department of Internal Medicine, Soonchunhyang University Cheonan Hospital, Cheonan, Republic of Korea; 8 Department of Internal Medicine, College of Medicine, Konyang University, Daejeon, Republic of Korea; 9 Division of Pulmonary and Critical Care Medicine, Department of Internal Medicine, Chungbuk National University College of Medicine, Cheongju, Republic of Korea; 10 Division of Pulmonary, Allergy, and Critical Care Medicine, Department of Internal Medicine, Yonsei University Wonju Severance Christian Hospital, Yonsei University Wonju College of Medicine, Wonju, Republic of Korea; 11 Division of Pulmonary and Critical Care Medicine, Department of Internal Medicine, Ilsan Paik Hospital, Inje University College of Medicine, 170 Juhwa-ro, Ilsanseo-gu, Goyang-si, Gyeonggi-do 10380 Republic of Korea; 12 Division of Pulmonary and Critical Care Medicine, Department of Internal Medicine, Incheon St. Mary’s Hospital, College of Medicine, The Catholic University of Korea, 56, Dongsu-ro, Incheon, 21431 Republic of Korea

**Keywords:** Microbiology, Diseases, Medical research

## Abstract

Disparities exist between sexes regarding tuberculosis (TB) incidence, as well as disease severity and outcome. Using a nationwide TB registry database, we explored the impact of sex and age on extrapulmonary TB (EPTB) among all enrolled patients by (1) calculating the female proportion for every age category according to TB-affected locations, (2) calculating the proportions of EPTB stratified by sex according to age, (3) conducting multivariable analysis to examine the impact of sex and age on EPTB likelihood, and (4) assessing the odds of EPTB for female compared to male as reference in every age category. Further, we explored the impact of sex and age on disease severity among pulmonary TB (PTB) patients. Of all the TB patients, 40.1% were female, with a male-to-female ratio of 1.49. The proportion of females was lowest in their fifties, resembling a U-shape. The male-to-female ratios in PTB and EPTB were 1.67 and 1.03, respectively. Compared to men, women were significantly associated with EPTB in their forties, fifties, and sixties. Female patients with PTB had significantly lower odds of having cavitation and positive smear test results in their fifties. Significant differences were found concerning TB location and severity between sexes, especially during reproductive age.

## Introduction

Tuberculosis (TB) is a major global public health problem that can affect any person, regardless of age or sex. However, disparities in notification rates between men and women have been observed for a long time. According to the World Health Organization’s 2007 report, the male-to-female ratio for the worldwide case notification rate was 1.96^1^. In some countries, this ratio may reach values as high as 3, and ratios below 1 are extremely rare. In 2020, the TB burden in adult men accounted for 56% of all cases; in comparison, adult women and children accounted for 33% and 11%, respectively^2^. There are huge concerns about the gender effect determined by socioeconomic and behavioral factors acting as barriers to health care access for women, particularly in developing countries, which might cause under-notification of female TB cases. However, considering the consistent reports about sex bias around the world, it is strongly suggested that biological sex differences do exist.

During the past decades, accumulating evidence has supported the impact of sex on disease pathogenesis, prevalence, severity, diagnosis, and treatment outcomes^3^. However, pathogenesis for development and progression of TB are not yet fully understood, and information regarding the underlying biological mechanisms of differential TB susceptibility according to sex is lacking. The aim of our study was to evaluate whether sex affects the clinical manifestations of active TB disease, such as disease location and severity, and whether the effect of sex varies across the life span.

## Materials and methods

### Study setting and participants: Korea TB cohort database

The Republic of Korea, a country with an intermediate-TB burden^4^, has been operating a public–private mix TB control project, in which approximately 70.7% of new patients with TB were notified and treated in 2018. All notified patients were followed until the end of treatment by TB specialist nurses dispatched to the participating hospitals^5^. We constructed a prospective observational registry database of the notified patients with TB included in this project, called the ‘Korea TB cohort database’^6^. During this period, data were systemically collected from patients notified to the national TB surveillance system by TB specialist nurses using a pre-specified questionnaire and case report form. After data gathering from local hospitals, a regional data manager organized the information every month and forwarded it to a central data manager on a quarterly basis. Central data managers in turn performed audits to control the quality of the registry. For this study, we retrieved data from the Korea TB cohort database between July 2018 and June 2019. Individuals positive for human immunodeficiency virus were excluded from the study population because of their very low rate in Korea.

### Variables

Baseline characteristics such as age, sex, body mass index (BMI), smoking and alcohol history, prior anti-TB treatment history, and co-existing comorbidities, were collected as independent variables. Age ranges were stratified into eight groups by 10-year increments (≤ 19, 20–29, 30–39, 40–49, 50–59, 60–69, 70–79, and 80–99). Malignancy was defined if diagnosed within 5 years of TB diagnosis.

The specific outcomes of interest were the development of extrapulmonary TB (EPTB) and initial disease severity. EPTB was defined according to WHO definitions^7^. We identified TB-affected organs among all patients with TB and encoded them as binary data: (1) pulmonary TB (PTB) with or without extrapulmonary involvement and (2) EPTB without clinical evidence of pulmonary involvement. To assess initial disease severity among patients with PTB, we chose four main outcome variables: presence of TB-related symptoms, chest radiographic findings of cavitation and bilateral infiltrations, and positive acid-fast bacilli (AFB) smear results. We collected TB-related symptoms using a predefined checklist that included cough, sputum, fever, general weakness, dyspnea, chest pain, body weight loss, and hemoptysis. If patients denied all the listed symptoms, they were regarded as asymptomatic.

### Statistical analysis

The baseline characteristics of all enrolled patients are presented as mean and standard deviation for continuous variables and as frequencies and percentages for categorical variables. Continuous variables were compared using t-test, and categorical variables were compared using chi-squared or Fisher’s exact test.

We undertook four analytical steps to explore the impact of sex and age on EPTB likelihood among all enrolled patients. First, we calculated the proportion of female sex individuals for each age category among enrolled patients with TB according to the location of the TB-affected organs. Second, we calculated the proportion of patients with EPTB stratified by sex in each of the defined age categories. Third, we assessed the odds of having EPTB for female sex compared to male sex individuals in every age category. The multivariable model was adjusted for age, sex, BMI, current smoking status, heavy alcohol intake, and presence of comorbidities. For the interaction analysis between age and sex, interaction term with sex and age was further added to the previous model, and subgroup analyses of the multivariable logistic regression model were performed in male and female patients separately. We followed the same analytical process to explore the impact of sex and age on the likelihood of each of the four indices of disease severity among patients with PTB. All statistical analyses were performed using R software (version 3.6.0).

### Ethics approval and consent to participate

This study adhered to the principles of the Declaration of Helsinki. The Institutional Review Board of Ilsan Paik Hospital, Inje University approved the study protocol (IRB No. ISPAIK 2021-08-012). The Korea Disease Control and Prevention Agency (KDCA), with the authority to hold and analyze surveillance data for public health and research purposes, approved the data use and provided data without personal identification information.

## Results

### Characteristic differences of female patients with TB

**Figure 1:**
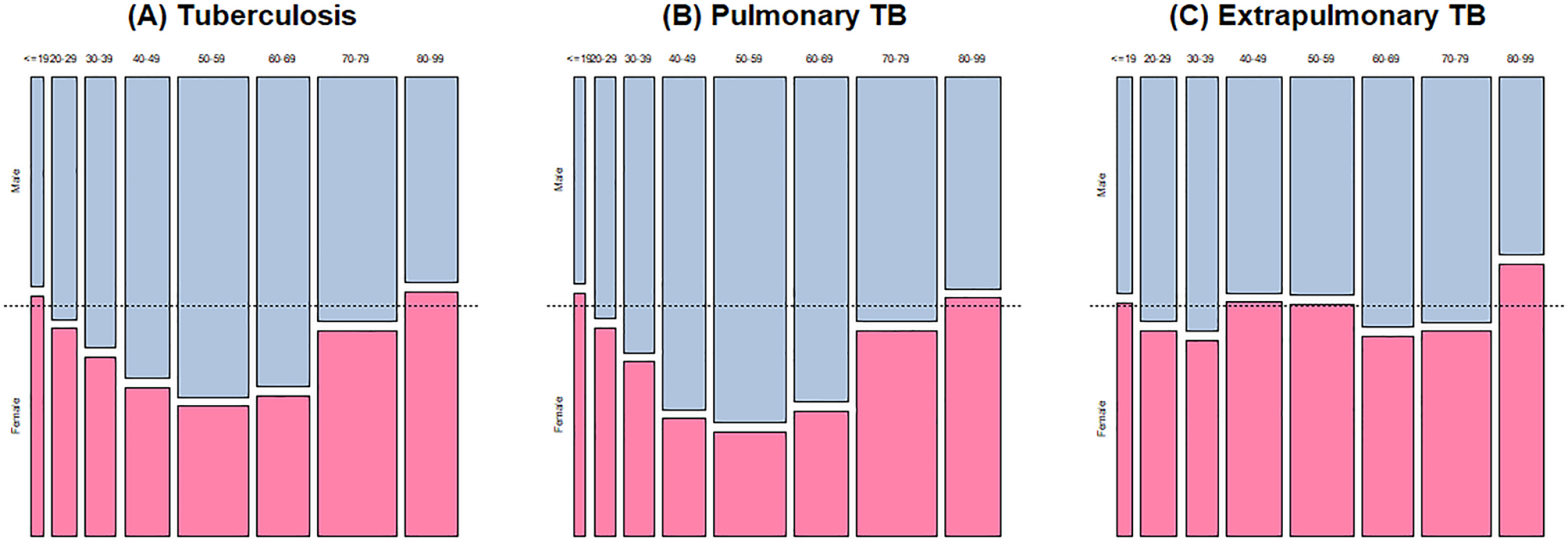
Changes of proportions of sex in 10-year age increments among (**A**) all the enrolled tuberculosis patients, (**B**) pulmonary tuberculosis patients, and (**C**) extrapulmonary tuberculosis patients. Areas of each box reflect relative number of patients in each group. *TB* tuberculosis.

A total of 5957 patients with TB were enrolled during the study period, and their baseline characteristics are summarized in Table 1. The mean age was 57.9 years old, and 2390 (40.1%) were female with a male to female ratio of 1.49. Compared to male patients, female patients were older (57.1 ± 18.4 vs. 59.1 ± 21.8, P < 0.001). The proportion of females in each age category serially decreased from the age group of ≤ 19 years (53.3%) reaching a bottom point at 50–59 years (28.9%) and then increasing up to 80–99 years (54.4%), resembling a U-shaped pattern (Fig. 1A). Female patients were less likely to be current smokers or heavy drinkers. Furthermore, the proportion of female patients with comorbidities was significantly lower, except for chronic neurologic disease. The frequency of symptoms was not different; however, extrapulmonary involvement was higher in female patients (18.8% vs. 27.3%, P < 0.001).

**Table 1:** Baseline characteristics of enrolled participants with tuberculosis stratified by sex.

Variables	Total (n = 5957)	Male (n = 3567)	Female (n = 2390)	P-value
Age, years	57.9 ± 19.8	57.1 ± 18.4	59.1 ± 21.8	< 0.001
Body mass index (kg/m^2^)	21.6 ± 3.5	21.7 ± 3.4	21.5 ± 3.5	0.024
Smoking status				< 0.001
Never or ex-smoker	4775 (80.2)	2470 (69.2)	2305 (96.4)	
Current smoker	1182 (19.8)	1097 (30.8)	85 (3.6)	
Drinking status				< 0.001
None or social drinker	5597 (93.1)	3229 (89.2)	2368 (99.0)	
Heavy drinker	360 (6.9)	338 (10.8)	22 (1.0)	
Comorbidity				0.002
Yes	3459 (58.1)	2129 (59.7)	1330 (55.7)	
No	2498 (41.9)	1438 (40.3)	1060 (44.3)	
Types of comorbidities				
Diabetes	1166 (19.6)	777 (21.8)	389 (16.3)	< 0.001
Chronic lung disease	277 (4.6)	194 (5.4)	83 (3.5)	0.001
Chronic heart disease	284 (4.8)	176 (4.9)	108 (4.5)	0.499
Chronic liver disease	127 (2.1)	97 (2.7)	30 (1.3)	< 0.001
Chronic kidney disease	198 (3.3)	138 (3.9)	60 (2.5)	0.005
Chronic neurologic disease	514 (8.6)	270 (7.6)	244 (10.2)	< 0.001
Malignancy	572 (9.6)	404 (11.3)	168 (7.0)	< 0.001
Autoimmune disease	67 (1.1)	33 (0.9)	34 (1.4)	0.097
Initial presenting symptoms				0.851
Yes	3901 (65.5)	2332 (65.4)	1569 (65.6)	
No	2056 (34.5)	1235 (34.6)	821 (34.4)	
Location of TB-affected organ				< 0.001
Pulmonary TB	4636 (77.8)	2898 (81.2)	1738 (72.7)	
Extrapulmonary TB	1321 (22.2)	669 (18.8)	652 (27.3)

The baseline characteristics of the patients with PTB and EPTB are described in Table 2. Among patients with PTB, women were older than men (60.5 ± 22.0 vs. 57.8 ± 17.9, P < 0.001); however, there were no age differences between females and males in patients with EPTB (55.4 ± 20.9 vs. 54.1 ± 20.1, P = 0.254). Male to female ratios in patients with PTB and EPTB were 1.67 and 1.03, respectively. The U-shaped pattern of sex proportions according to age categories was still observed in the PTB group (Fig. 1B), similar to the entire population; however, this pattern was not observed in the EPTB group (Fig. 1C). However, the graph presenting female sex proportions across the age categories in TB pleurisy also resembled the U-shaped pattern present in PTB (Supplemental Fig. 1). In patients with TB lymphadenitis, the proportion of female patients was persistently higher than that of male patients, except for the groups—20–29 and 30–39. The proportions of current smoking status and heavy alcohol intake were significantly lower among female patients, regardless of TB site (Table 2). Among patients with EPTB, the proportion of patients with any chronic diseases and having any related symptoms was lower in female patients.

**Table 2:** Baseline characteristics of enrolled participants with pulmonary and extrapulmonary tuberculosis stratified by sex.

Variables	Pulmonary tuberculosis				Extrapulmonary tuberculosis			
	Total (n = 4636)	Male (n = 2898)	Female (n = 1738)	P-value	Total (n = 1321)	Male (n = 669)	Female (n = 652)	P-value
Age, years	58.8 ± 19.6	57.8 ± 17.9	60.5 ± 22.0	< 0.001	54.8 ± 20.5	54.1 ± 20.1	55.4 ± 20.9	0.254
Body mass index (kg/m^2^)	21.3 ± 3.4	21.4 ± 3.3	21.1 ± 3.4	0.038	22.7 ± 3.6	23.1 ± 3.4	22.4 ± 3.7	< 0.001
Smoking status				< 0.001				< 0.001
Current smoker	1016 (21.9)	948 (32.7)	68 (3.9)		166 (12.6)	149 (22.3)	17 (2.6)	
Drinking status				< 0.001				< 0.001
Heavy drinker	312 (7.6)	294 (11.5)	18 (1.2)		48 (4.2)	44 (7.7)	4 (0.7)	
Comorbidity				0.084				0.010
Yes	2738 (59.1)	1740 (60.1)	998 (57.5)		721 (54.6)	389 (58.1)	332 (50.9)	
Types of comorbidities								
Diabetes	956 (20.6)	652 (22.5)	304 (17.5)	< 0.001	210 (15.9)	125 (18.7)	85 (13.0)	0.006
Chronic lung disease	238 (5.1)	164 (5.7)	74 (4.3)	0.043	39 (3.0)	30 (4.5)	9 (1.4)	0.002
Chronic heart disease	221 (4.8)	138 (4.8)	83 (4.8)	> 0.999	63 (4.8)	38 (5.7)	25 (3.8)	0.149
Chronic liver disease	96 (2.1)	75 (2.6)	21 (1.2)	0.002	31 (2.3)	22 (3.3)	9 (1.4)	0.035
Chronic kidney disease	130 (2.8)	91 (3.1)	39 (2.2)	0.090	68 (5.1)	47 (7.0)	21 (3.2)	0.003
Chronic brain disease	396 (8.5)	207 (7.1)	189 (10.9)	< 0.001	118 (8.9)	63 (9.4)	55 (8.4)	0.597
Malignancy	456 (9.8)	341 (11.8)	115 (6.6)	< 0.001	116 (8.8)	63 (9.4)	53 (8.1)	0.465
Autoimmune disease	53 (1.1)	27 (0.9)	26 (1.5)	0.108	14 (1.1)	6 (0.9)	8 (1.2)	0.751
Initial presenting symptoms				0.851				0.004
Yes	2580 (65.5)	1663 (65.4)	917 (65.6)		952 (72.1)	506 (75.6)	446 (68.4)	
Chest radiograph findings								
Cavitation	869 (19.2)	651 (23.0)	218 (12.9)	< 0.001				
Bilateral infiltrations	1435 (32.7)	955 (34.6)	480 (29.5)	0.001				
Sputum AFB test results								
AFB smear positivity	1182 (29.3)	793 (31.0)	389 (26.3)	0.002				
AFB culture positivity	2422 (60.9)	1538 (61.0)	884 (60.8)	0.902				

*AFB* acid-fast bacilli.

### Female patients and EPTB development

**Figure 2:**
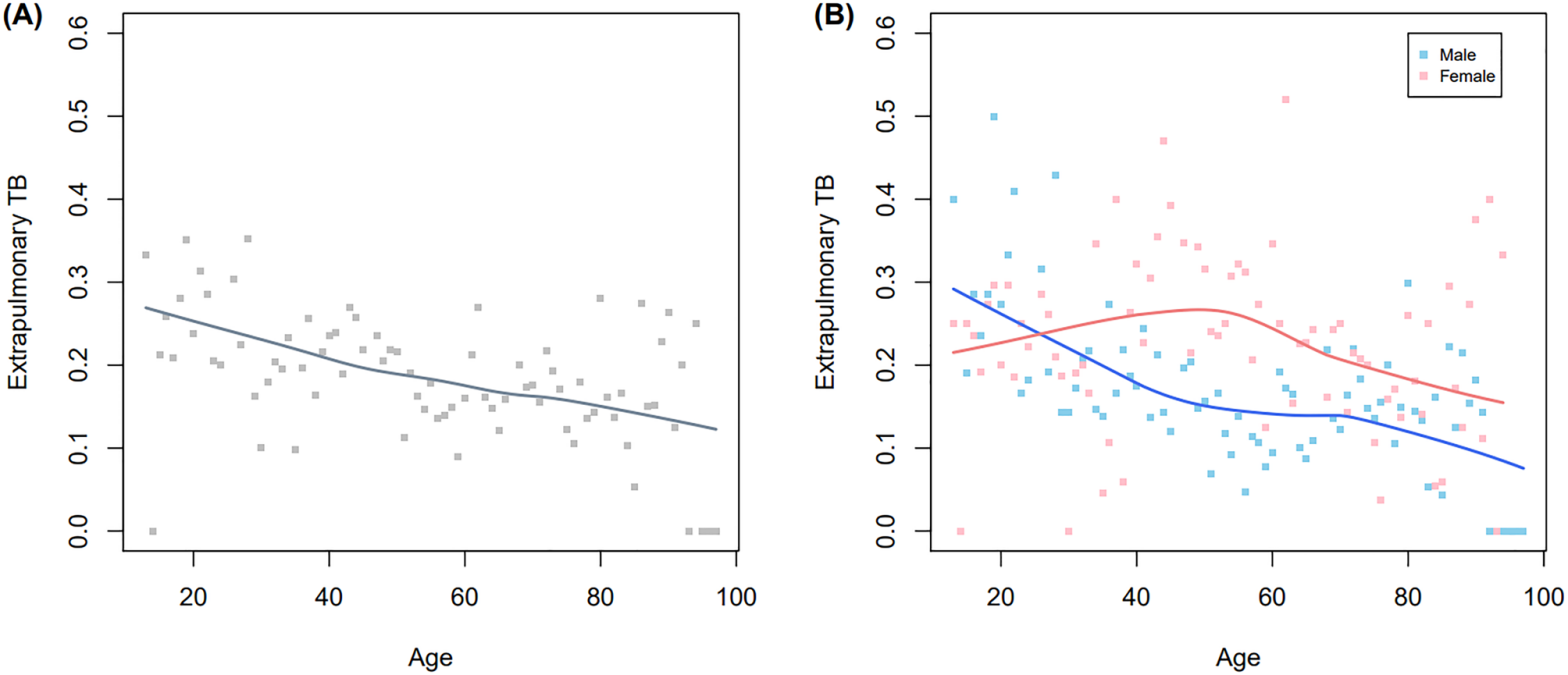
Association of sex and extrapulmonary tuberculosis among all the enrolled tuberculosis patients: changes of proportions of extrapulmonary tuberculosis according to age (**A**), stratified by sex (**B**). *TB* tuberculosis.

**Figure 3:**
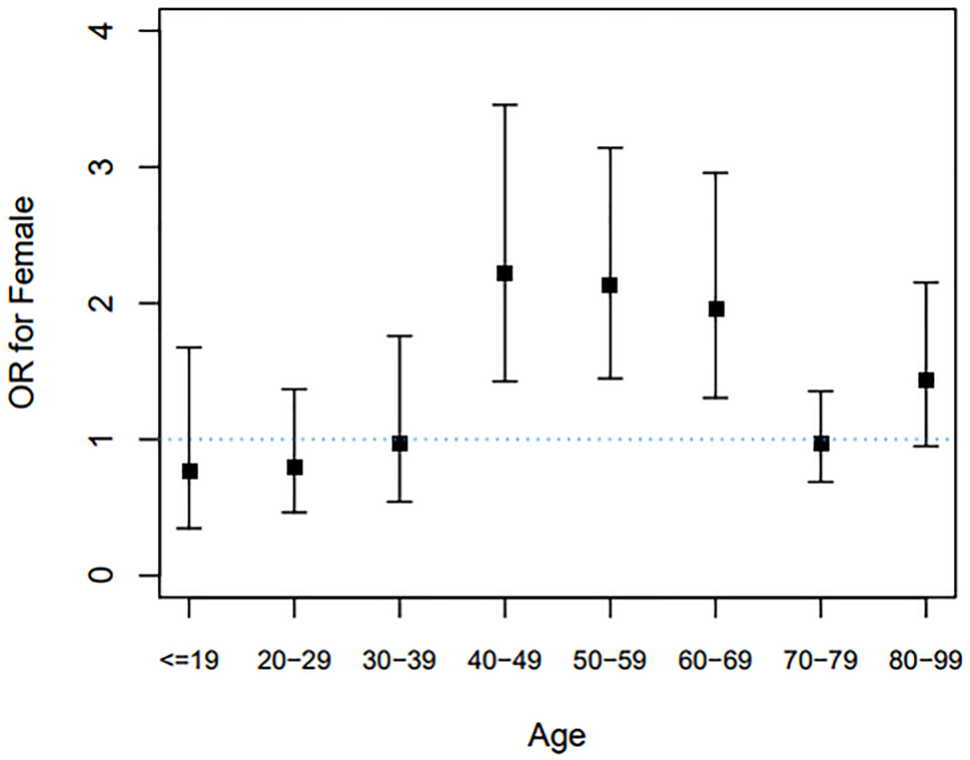
Multivariable analysis showing odds of extrapulmonary tuberculosis for female versus male in 10-year age increments. We assessed the odds of having extrapulmonary tuberculosis for females compared with males as a reference in every age category. The multivariable model was adjusted for age, sex, body mass index, current smoking status, heavy alcohol intake, and the presence of comorbidities. *OR* odds ratio.

Regarding the association between EPTB and age, we observed that EPTB prevalence decreased with increasing age (Fig. 2A). Further, this association was stratified by sex. The proportion of female patients with EPTB was lower than that of male among teenagers (≤ 19 age group) (Fig. 2B). However, this proportion increased in 20–29 and 30–39 age groups and the higher EPTB prevalence was maintained for female patients after their forties. To assess the association between age, sex, and EPTB development, multivariable logistic regression analyses were performed. The odds ratios (OR) of age and female sex for EPTB development were 0.992 (95% CI 0.988–0.996) and 1.481 (95% CI 1.280–1.713), respectively. Multivariable analyses of the effect of female sex on EPTB development were stratified according to age category (Fig. 3, Table 3). Compared to male patients, female patients were significantly associated with EPTB in the following age groups: 40–49 (adjusted OR [aOR] 2.219, 95% CI 1.425–3.456), 50–59 (aOR 2.132, 95% CI 1.447–3.140), and 60–69 (aOR 1.964, 95% CI 1.304–2.957). However, there was not a statistically significant interaction between age and sex for EPTB due to non-linear relationship (P = 0.944).

**Table 3:** Effect of female sex compared to male sex on extrapulmonary tuberculosis according to age category.

Age categories (years)	Univariable analysis OR (95% CI)	Multivariable analysis OR (95% CI)
≤ 19	0.835 (0.441–1.580)	0.762 (0.347–1.675)
20–29	0.788 (0.501–1.240)	0.797 (0.464–1.369)
30–39	0.960 (0.604–1.527)	0.976 (0.542–1.759)
40–49	2.521 (1.767–3.597)	2.219 (1.425–3.456)
50–59	2.853 (2.044–3.981)	2.132 (1.447–3.140)
60–69	2.117 (1.481–3.026)	1.964 (1.304–2.957)
70–79	1.022 (0.753–1.387)	0.964 (0.687–1.354)
80–99	1.243 (0.868–1.781)	1.430 (0.950–2.152)

The multivariable model was adjusted for age, sex, body mass index, current smoking status, heavy alcohol intake, and the presence of comorbidities.

*OR* odds ratio, *CI* confidence interval.

### Female patients and disease severity in PTB

Among patients with PTB, women showed lower proportions of cavitary disease (23.0% vs. 12.9%, P < 0.001), bilateral disease (34.6% vs. 29.5%, P = 0.001), and positive AFB smear test results (31.0% vs. 26/3%, P = 0.002) than men (Table 2). The proportions of female patients that had initial presenting symptoms, cavitary disease, bilateral disease, and AFB smear positivity were lowest in the 50–59 age category, also resembling a U-shaped pattern (Supplemental Fig. 2).

Among patients with PTB, age was positively associated with the presence of initial presenting symptoms, bilateral disease, and AFB smear positivity but negatively associated with cavitary disease in multivariable analysis (Supplementary Table 1). Associations between sex and symptom presence, bilateral disease, and AFB smear positivity were not significant. However, a significant association between sex and for cavitary disease (aOR 0.661, 95% CI 0.544–0.803) was found. The interactions between sex and age for each disease severity index were not significant because of non-linear associations.

**Figure 4:**
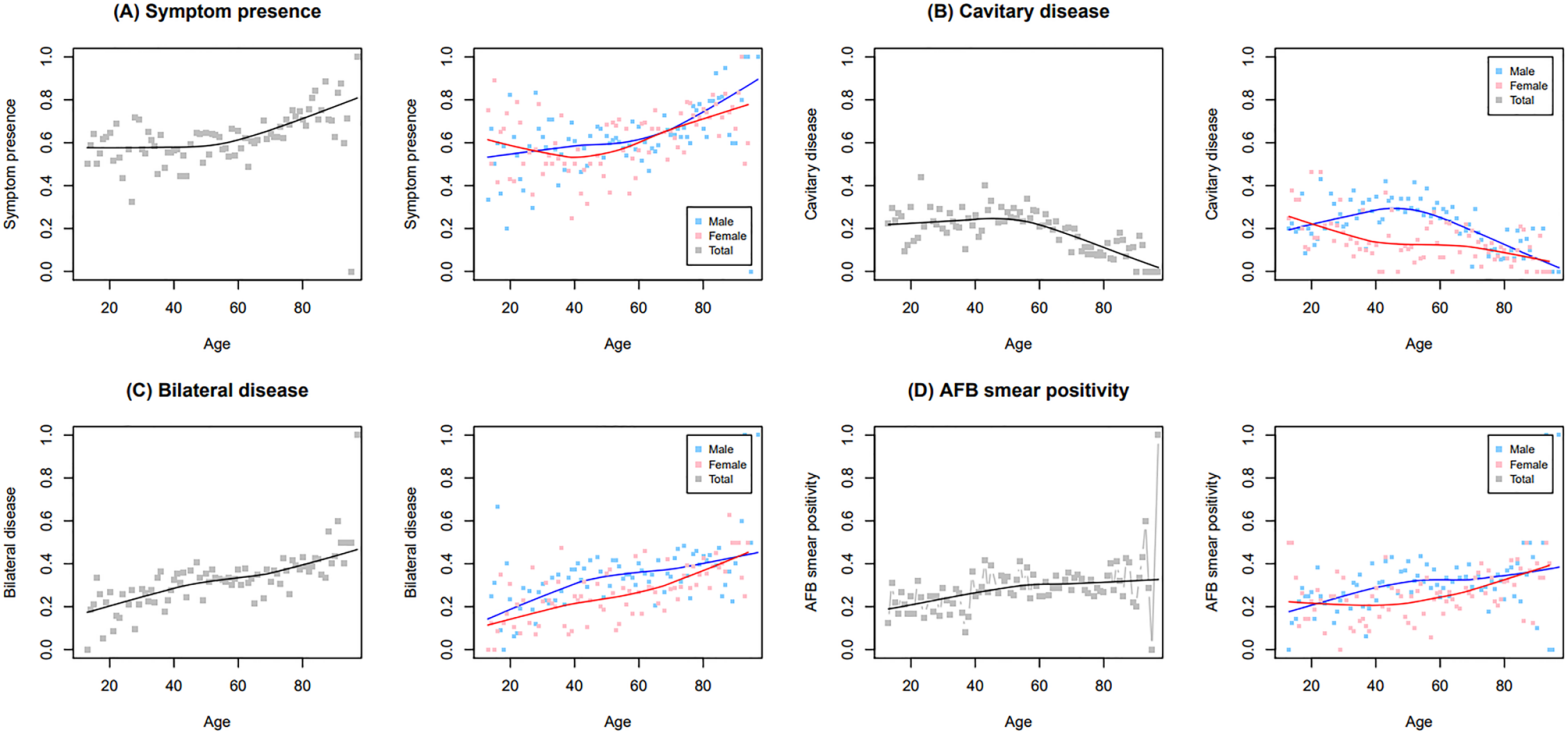
Changes of proportions of disease severity of interest according to age stratified by sex among pulmonary tuberculosis patients: (**A**) symptom presence, (**B**) cavitary disease, (**C**) bilateral disease, and (**D**) AFB smear positivity. *AFB* acid-fast bacilli.

**Figure 5:**
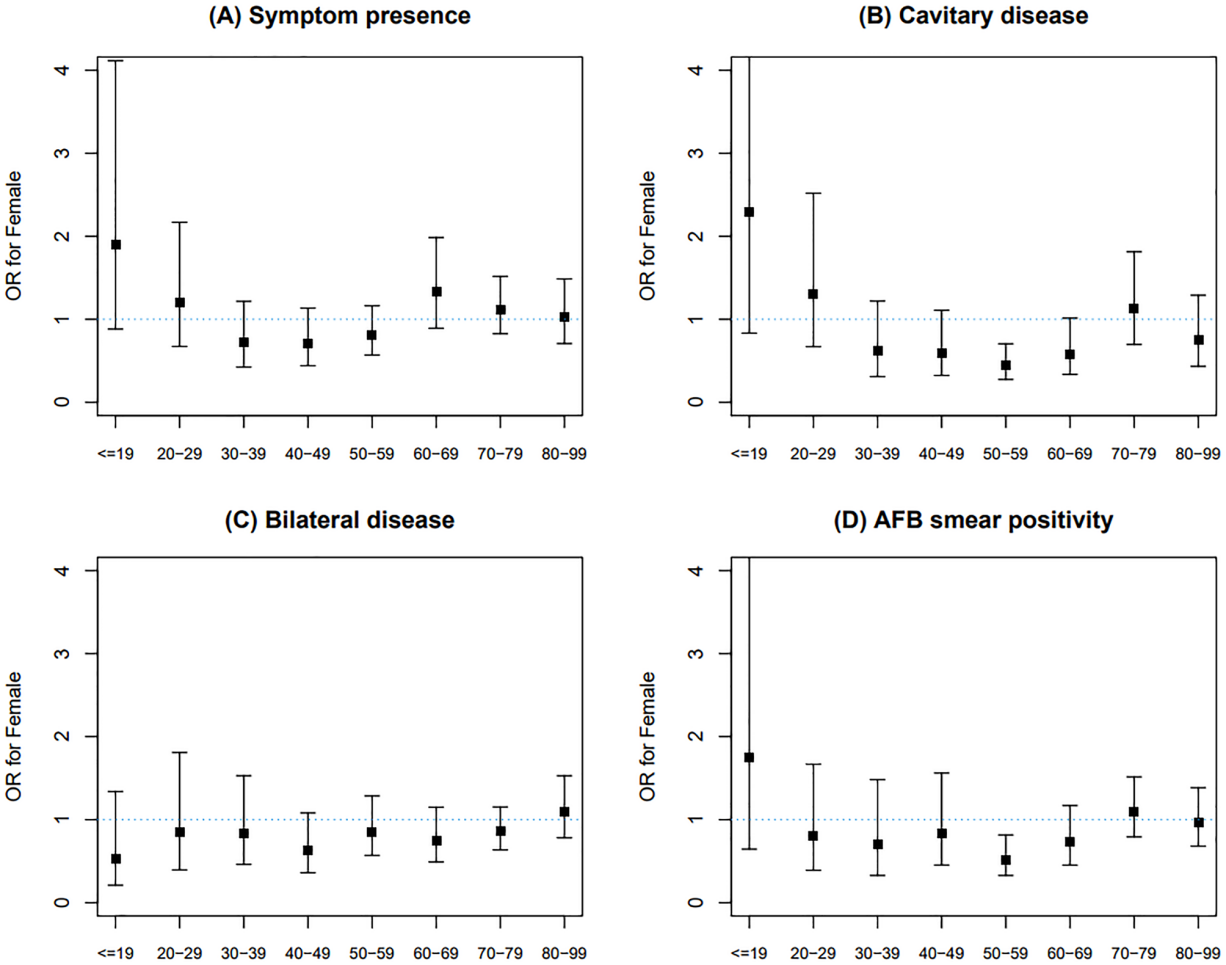
Multivariable analysis showing odds of disease severity of interest for female versus male in 10-year age increments: (**A**) symptom presence, (**B**) cavitary disease, (**C**) bilateral disease, and (**D**) AFB smear positivity. We assessed the odds of having extrapulmonary tuberculosis for females compared with males as a reference in every age category. The multivariable model was adjusted for age, sex, body mass index, current smoking status, heavy alcohol intake, and the presence of comorbidities. *OR* odds ratio.

In male patients with PTB, the proportions of symptom presence, bilateral disease, and AFB smear positivity increased linearly with age, whereas in female patients the curve showed a dimpling point (Fig. 4). The proportion of cavitary disease decreased linearly as age increased in female patients; however, its distribution in male patients followed a convex pattern with the highest value around their forties. We also conducted univariable and multivariable analyses to assess the association between female sex and disease severity of interest for each age category (Supplemental Table 2). The results of univariable analysis showed concave patterns centered around the age group of 40–49 for all indices of disease severity (Fig. 5). In the univariable analysis, females were less likely to have initial presenting symptoms in their forties; however, this association was attenuated in the multivariable analysis (Fig. 5A). Female patients were also less likely to have cavitary disease in their thirties, forties, fifties, and sixties in the univariable analysis; however, their odds for this condition only remained significant for the 50–59 age group (aOR 0.440, 95% CI 0.275–0.704) in the multivariable analysis (Fig. 5B). Although females in their forties and fifties were less likely to have bilateral disease in the univariable analysis, differences in the odds corresponding to these age groups were not detected in the multivariable analysis (Fig. 5C). In the univariable analysis, differences in the odds of having positive AFB smear results between sexes were detected in the 50–59 and 60–69 age groups; however, only females in their fifties showed significance for AFB smear positivity in the multivariable analysis (aOR 0.517, 95% CI 0.327–0.816) (Fig. 5D).

## Discussion

Based on a large nationwide registry database in Korea, we confirmed that the initial clinical presentations at the time of TB diagnosis were different between male and female sexes. First, female patients with TB were more likely to have extrapulmonary involvement, and the differences were statistically significant between their forties and sixties. Second, female patients had milder forms of PTB, especially between their thirties and fifties. The proportions of cavitation on chest radiography and positive smear test results among female patients with PTB were significantly lower in their fifties, compared to male patients. The results of our analysis concerning the different epidemiological characteristics between sexes would guide both clinicians and public health officers to prepare individualized diagnostic and therapeutic strategies according to sex.

Over the past decades, developed countries have witnessed an increase in the average age of conception and delivery for women^8^. In Korea, the proportion of pregnancies after 35 years of age increased from 3.9% in 1981 to 33.4% in 2019^9^. During pregnancy, the diagnosis of TB is frequently delayed due to non-specific symptoms and their masking by pregnancy symptoms. Our results showed that women in their thirties had milder forms of PTB and those in their forties were more likely to have extrapulmonary involvement, which suggests that it would be more challenging to diagnose TB in older pregnant women in a timely manner. Maternal health services often neglect TB services for pregnant women and their families. Maternal and neonatal health programs should collaborate more to maximize the access to TB care for women. In addition, social and psychological supports against TB-related stigma are also necessary to protect women during the postpartum period with a higher prevalence of depressive episodes.

TB infection usually begins in the respiratory tract. After primary pulmonary infection, hematogenous and lymphatic spreads of *Mycobacterium tuberculosis* bacilli can occur, which can be ascribed to insufficient immune responses to confine the bacilli to the lung parenchyma. However, the pathogenesis of the disease development and progression of TB is still not fully understood. Several studies have investigated the impact of sex on the occurrence of EPTB and found that women were more likely to present with active TB as EPTB^10–12^, which is consistent with our findings.

Both genetic factors and hormonal mediators could account for sex-based disparities in immune responses^13^. Notably, we found a higher likelihood of extrapulmonary involvement in female patients between their forties and sixties, which suggests the effect of hormonal mediators. A previous Taiwanese study^10^ showed that among patients with PTB aged 45 years or older, women were more likely to have concurrent EPTB. They speculated that the immune system of older women was less able to contain bacilli locally in the lung parenchyma. It is generally perceived that estradiol functions as an immunity-sustaining or immunity-enhancing mediator and testosterone works as a mediator that inhibits the immune response. Women’s reproductive ages ranged from 15 to 49 years. During perimenopause, which usually occurs in their forties, estrogen levels begin to decline, which could unbalance women’s immune response to confine bacilli and release them through the hematologic and lymphatic systems. When treating women in their forties and sixties with suspected TB, it is necessary to evaluate the simultaneous presence of EPTB using appropriate diagnostic tools.

We chose four indices to assess sex differences in disease severity among patients with PTB and found that women had a milder form of the disease, especially in their thirties and fifties. It has been reported that men are more prone to be infected with TB and are associated with a more severe clinical presentation and poorer outcomes^11,14–16^. This sex imbalance may be attributable to a complex mixture of biological, epidemiological, and sociocultural determinants. There are several biological ways to explain sex as a modifier of disease. Females exhibit robust immune responses to antigenic challenges, such as infection and vaccination, which are largely mediated by sex hormones^17^. Sex hormones have diverse effects on many immune cell types, including B/T lymphocytes, neutrophils, dendritic cells, macrophages, and natural killer cells, which are key players in TB-related immune responses^1^. Furthermore, apart from sex hormones, females could benefit from advantageous genetic diversity through cellular mosaicism and genes escaping X chromosome inactivation, as a result of having two X chromosomes^18^. Additionally, immune-related genes on the X chromosome can affect TB-related immunity^19^.

Behavioral and socio-cultural determinants have also been proposed to explain sex bias in TB. Gender can affect the development and progression of TB owing to differences in social roles, risk behaviors, and activities. Dependence on alcohol and tobacco is generally higher in men, which aggravates the initial clinical presentation at the time of TB diagnosis. Both alcohol abuse^20^ and smoking^21^ are associated with disease progression, disease severity, and poor treatment outcomes among patients with TB. Therefore, we aimed to adjust for these effects in multivariable analysis in order to exclude the potential influence of men’s deleterious health behaviors. According to our multivariable logistic regression analysis, the proportions of cavitation on chest radiography and positive smear test results among female patients with PTB in their fifties were significantly lower, in accordance with previous studies^15,22^.

In addition, women are more likely to submit poor-quality specimens than men, which might cause a low-grade smear test result^23^; however, this does not explain the lesser cavitary and bilateral diseases at initial diagnosis. Another explanation for milder disease forms in women is that TB diagnosis is made earlier in young women who can visit clinics more frequently during childbearing ages, which could increase the early detection of TB disease thanks to various chances of TB screening^11^.

TB has heterogeneous phenotypes, which makes it challenging to diagnose^6^. Males and females with TB present with distinct clinical phenotypes despite having the same TB disease. However, our understanding of sex differences regarding these heterogeneities is still lacking. If males and females differ in their inflammatory properties during TB infection, these differences must be considered when developing and implementing new therapeutic approach^24^. Identifying sex-specific biomarkers is necessary to understand the different TB pathophysiology in males and females and to facilitate the development of more effective diagnostic and therapeutic methods for TB infection.

The key strength of our study is the analysis of a large number of notified TB cases systemically collected across the country, which can represent the actual burden of female and male TB patients in Korea. Thus, it can be used as a document by policymakers to prioritize curative and preventive action plans. However, there are several limitations that should be acknowledged. We could not evaluate the effects of sex disparities on treatment outcomes. Additional follow-up of our cohort is required to determine specific treatment outcomes. Because we used the nationwide TB cohort database, which was primarily built for the notification of TB cases in Korea, we were unable to collect additional clinical information. For example, laboratory findings, such as blood cell counts, C-reactive protein, and interferon-gamma levels, were unavailable. We were also unable to collect detailed medication histories, such as chemotherapy and disease-modifying anti-rheumatic drugs. However, having malignancy and autoimmune disease, which were available in our database and comprised those who received such medications, might be indictive of chemotherapy and immunosuppressants. Various social determinants of health may have different effects on TB disease across the life span; however, we were unable to assess the influences of other health disparity indices, such as socio-economic status, on TB. Alongside the global trend of an aging population, elderly TB is posing a significant public health burden. Further research is necessary to understand the complex linkage of other health disparity indices and TB disease among elderly populations compared to younger populations.

## Conclusion

We have demonstrated that there are sex disparities in the clinical manifestations of TB across lifespan. The results of this study indicate that special attention should be paid to middle-aged men who are prone to present with severe forms of PTB at the initial diagnosis, which promotes active case findings and prompt anti-TB treatment. Our results also indicate that middle-aged women with TB should be carefully investigated for the presence of concurrent EPTB, which requires high suspicion and appropriate diagnostic tools. TB is a health issue with significant sex-related disparities that are not always widely recognized^25^. Our findings set a path for future research to understand different TB pathogenesis between sexes, to develop sex-targeted clinical assessment and management approaches for precision medicine, and to implement a more effective national TB control program.

## Supplementary Information

Supplementary Information.

## Data Availability

The ownership of the primary datasets lies with the Korea Disease Control and Prevention Agency (KDCA). The datasets generated and/or analyzed during the current study are available from the corresponding author on reasonable request.
