# Supplementary material for: Differential effects of sex on tuberculosis location and severity across the lifespan
Source: Sci Rep. 2023 Apr 13;13:6023. doi: 10.1038/s41598-023-33245-5 (PMC10102118; doi:10.1038/s41598-023-33245-5)
Supplement: Supplementary file 1 — Supplementary Information. [file 41598_2023_33245_MOESM1_ESM.pdf]

Differential effects of sex on tuberculosis location and severity across the lifespan

Author

Jinsoo Min[1,12], Jae Seuk Park[2,12], Hyung Woo Kim[3], Yousang Ko[4], Jee Youn Oh[5], Yun-Jeong Jeong[6], Ju Ock Na[7], Sun-Jung Kwon[8], Kang Hyeon Choe[9], Won-Yeon Lee[10], Sung-Soon Lee[11], Ju Sang Kim[3,13], and Hyeon-Kyoung Koo[11,13]

Affiliation

[1] Division of Pulmonary and Critical Care Medicine, Department of Internal Medicine, Seoul St. Mary's Hospital, College of Medicine, The Catholic University of Korea, Seoul, Republic of Korea

[2] Division of Pulmonary Medicine, Department of Internal Medicine, Dankook University College of Medicine, Cheonan, Republic of Korea

[3] Division of Pulmonary and Critical Care Medicine, Department of Internal Medicine, Incheon St. Mary's Hospital, College of Medicine, The Catholic University of Korea, Seoul, Republic of Korea

[4] Division of Pulmonary, Allergy and Critical Care Medicine, Department of Internal Medicine, Kangdong Sacred Heart Hospital, Hallym University College of Medicine, Seoul, Republic of Korea

[5] Division of Pulmonary, Allergy, and Critical Care Medicine, Department of Internal Medicine, Korea University Guro Hospital, Korea University College of Medicine, Seoul, Republic of Korea

[6] Division of Pulmonary and Critical Care Medicine, Department of Internal Medicine, Dongguk University Ilsan Hospital, Dongguk University College of Medicine, Goyang, Republic of Korea

[7] Department of Internal Medicine, Soonchunhyang University Cheonan Hospital, Cheonan, Republic of Korea

[8] Department of Internal Medicine, College of Medicine, Konyang University, Daejeon, Republic of Korea

[9] Division of Pulmonary and Critical Care Medicine, Department of Internal Medicine, Chungbuk National University College of Medicine, Cheongju, Republic of Korea.

[10] Division of Pulmonary, Allergy, and Critical Care Medicine, Department of Internal Medicine, Yonsei University Wonju Severance Christian Hospital, Yonsei University Wonju College of Medicine, Wonju, Republic of Korea

[11] Division of Pulmonary and Critical Care Medicine, Department of Internal Medicine, Ilsan Paik Hospital, Inje University College of Medicine, Goyang, Republic of Korea

Supplements

Supplemental Table 1. Association of age and sex with disease severities of pulmonary tuberculosis, interaction between age and sex, and main effect of age stratified by sex

	Age	Sex	Age*Sex		Male (age)	Female (age)
	OR (95% CI)	OR (95% CI)	Coef	P-value	OR (95% CI)	OR (95% CI)
Symptom presence	1.013 (1.009-1.017)	1.079 (0.934-1.247)	0.003	0.423	1.011 (1.006-1.016)	1.014 (1.008-1.020)
Cavitary disease	0.985 (0.980-0.989)	0.661 (0.544-0.803)	-0.001	0.754	0.985 (0.979-0.991)	0.986 (0.978-0.993)
Bilateral disease	1.013 (1.009-1.017)	0.876 (0.752-1.020)	0.007	0.083	1.011 (1.005-1.017)	1.014 (1.008-1.021)
AFB smear positivity	1.009 (1.005-1.013)	0.853 (0.723-1.005)	0.004	0.277	1.007 (1.001-1.013)	1.009 (1.002-1.016)

OR, odds ratio; CI, confidence interval; Coef, coefficient; AFB, acid-fast bacilli.

The multivariable model was adjusted for age, sex, body mass index, current smoking status, heavy alcohol intake, and the presence of comorbidities.

Supplemental Table 2. Effect of female sex compared to male sex on disease severity of interest in pulmonary tuberculosis: (A) symptom presence, (B) cavitary disease, (C) bilateral disease, and (D) AFB smear positivity

(A) Presence of symptoms

Age categories, years	Univariable analysis		Multivariable analysis	
	cOR	95% CI	aOR	95% CI
≤19	1.949	1.010-3.761	1.906	0.883-4.114
20-29	0.951	0.587-1.541	1.208	0.673-2.168
30-39	0.763	0.503-1.157	0.718	0.424-1.217
40-49	0.630	0.427-0.931	0.707	0.441-1.134
50-59	0.867	0.634-1.186	0.814	0.569-1.163
60-69	1.209	0.854-1.713	1.331	0.893-1.985
70-79	1.071	0.820-1.399	1.119	0.826-1.517
80-99	0.991	0.703-1.397	1.026	0.708-1.487

(B) Cavitary disease.

Age categories, years	Univariable		Multivariable	
	cOR	95% CI	aOR	95% CI
≤19	1.102	0.501-2.422	2.288	0.833-6.283
20-29	0.981	0.563-1.710	1.299	0.670-2.519
30-39	0.457	0.264-0.790	0.614	0.309-1.220
40-49	0.373	0.219-0.636	0.599	0.323-1.109
50-59	0.355	0.231-0.546	0.440	0.275-0.704
60-69	0.468	0.290-0.756	0.584	0.336-1.014

70-79	0.866	0.572-1.311	1.124	0.697-1.813
80-99	0.685	0.414-1.135	0.748	0.433-1.290

(C) Bilateral disease

Age categories, years	Univariable analysis		Multivariable analysis	
	cOR	95% CI	aOR	95% CI
≤19	0.638	0.281-1.445	0.530	0.210-1.339
20-29	0.974	0.520-1.827	0.844	0.394-1.809
30-39	0.807	0.497-1.312	0.940	0.461-1.529
40-49	0.540	0.339-0.860	0.624	0.361-1.081
50-59	0.640	0.445-0.922	0.855	0.569-1.285
60-69	0.730	0.496-1.073	0.750	0.489-1.150
70-79	0.748	0.573-0.977	0.856	0.636-1.152
80-99	0.981	0.717-1.341	1.092	0.780-1.529

(D) AFB smear positivity

Age categories, years	Univariable analysis		Multivariable analysis	
	cOR	95% CI	aOR	95% CI
≤19	1.485	0.632-3.487	1.747	0.645-4.735
20-29	0.970	0.520-1.818	0.806	0.389-1.667
30-39	0.575	0.324-1.021	0.696	0.327-1.481
40-49	0.650	0.401-1.054	0.840	0.452-1.561
50-59	0.509	0.338-0.765	0.517	0.327-0.816
60-69	0.644	0.421-0.984	0.727	0.452-1.170

70-79	0.961	0.717-1.287	1.095	0.792-1.515
80-99	1.018	0.731-1.416	0.970	0.680-1.384

cOR, crude odds ratio; CI, confidence interval; aOR, adjusted odds ratio; AFB, acid-fast bacilli.

The multivariable model was adjusted for age, sex, body mass index, current smoking status, heavy alcohol intake, and the presence of comorbidities.

Supplemental Figure 1. Changes in the proportions of sex in 10-year age increments for (A) TB pleurisy, (B) TB lymphadenopathy, (C) intestinal TB, (D) bone/joint TB, (E) central nervous system TB, and (F) genitourinary TB.

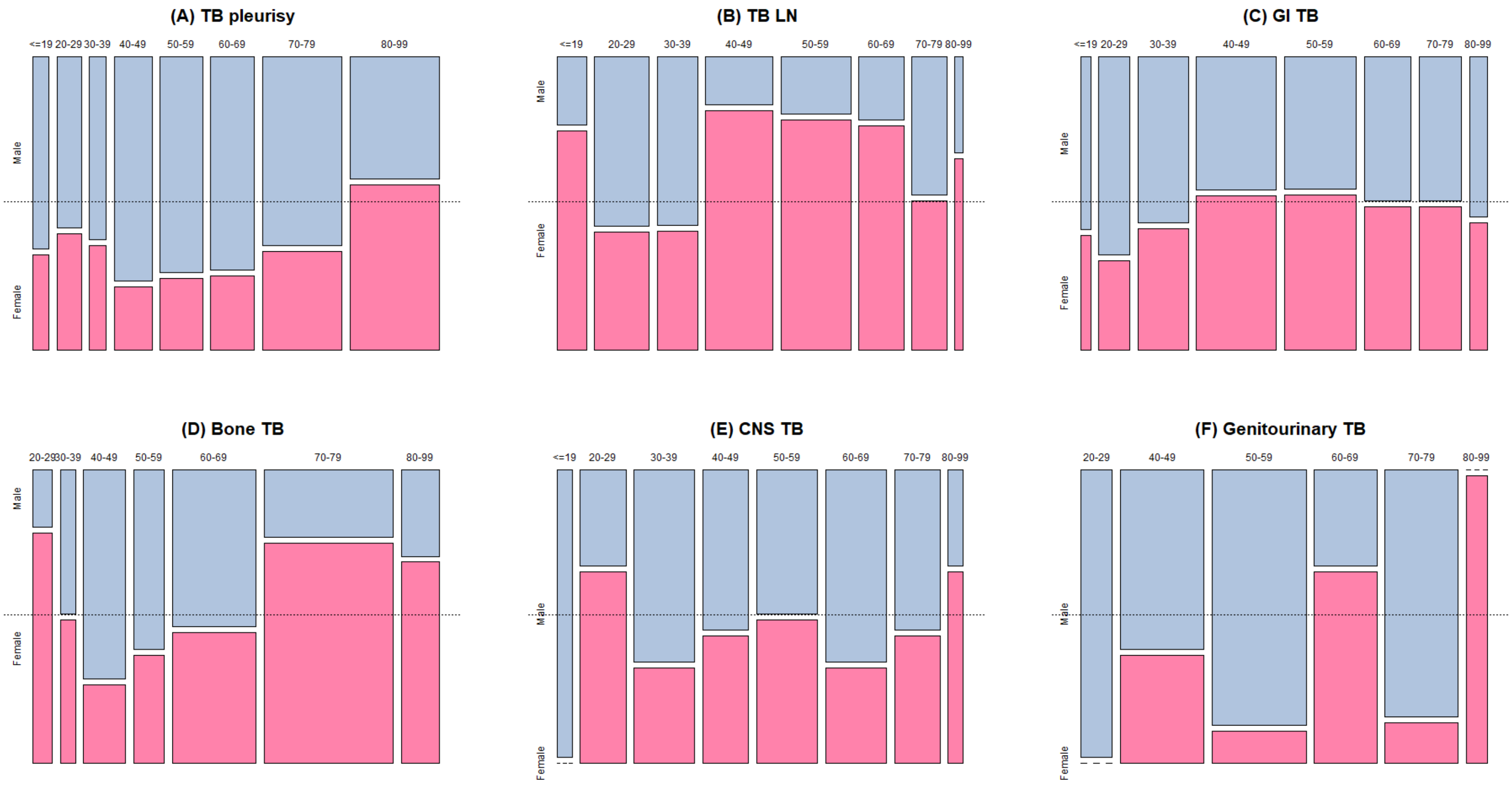

TB, tuberculosis

Areas of each box reflect relative number of patients in each group.

Supplemental Figure 2. Changes in proportions of sex in 10-year age increments among patients with pulmonary tuberculosis for each disease severity of interest: (A) symptom presence, (B) cavitary disease, (C) bilateral disease, and (D) AFB smear positivity

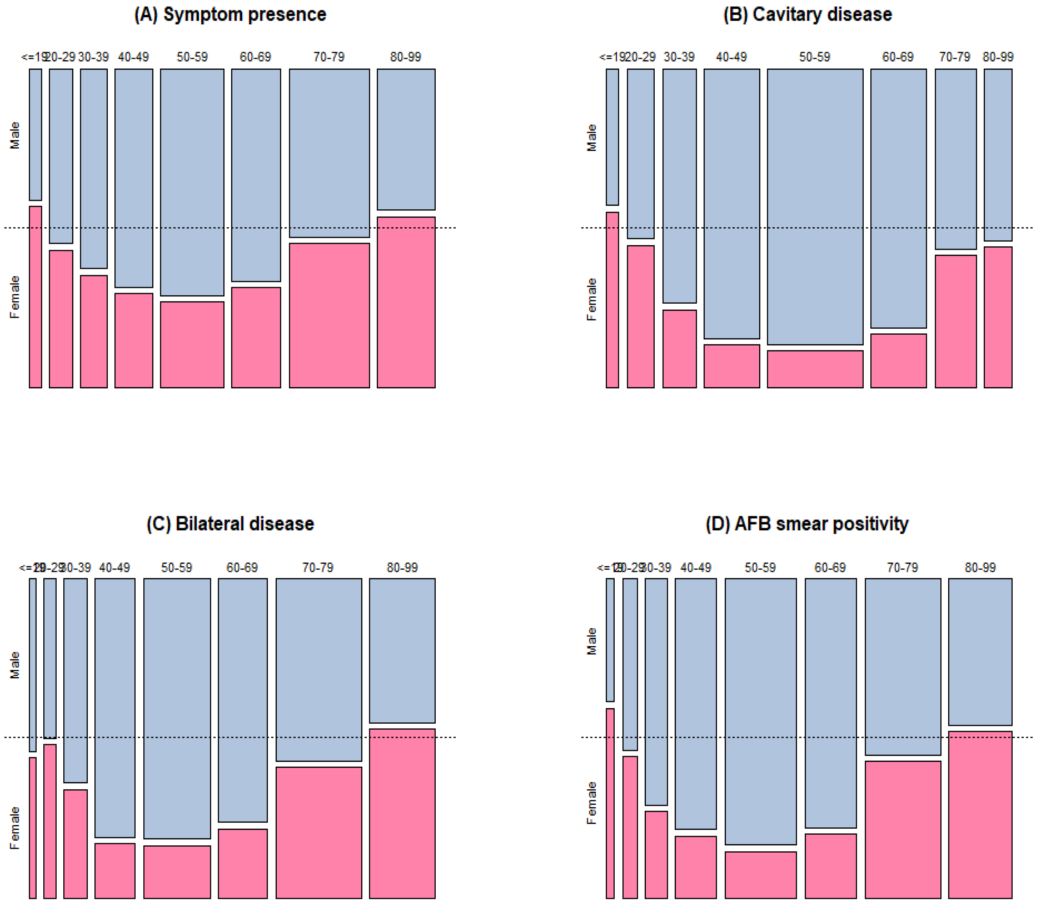

AFB, acid-fast bacilli

Areas of each box reflect relative number of patients in each group.
